# A novel retroviral mutagenesis screen identifies prognostic genes in RUNX1 mediated myeloid leukemogenesis

**DOI:** 10.18632/oncotarget.5133

**Published:** 2015-09-12

**Authors:** Dustin T. Rae, Jonah D. Hocum, Victor Bii, H. Joachim Deeg, Grant D. Trobridge

**Affiliations:** ^1^ Washington State University College of Pharmacy, Spokane, WA, USA; ^2^ Clinical Research Division, Fred Hutchinson Cancer Research Center, Seattle, WA, USA; ^3^ School of Molecular Biosciences, Washington State University, Pullman, WA, USA

**Keywords:** Chromosome Section, myelodysplastic syndrome, acute myeloid leukemia, mutagenesis screen, biomarker

## Abstract

Using a novel retroviral shuttle vector approach we identified genes that collaborate with a patient derived RUNX1 (AML1) mutant. RUNX1 mutations occurs in 40% of myelodysplastic syndromes (MDS). MDS are a group of hematopoietic stem cell disorders that are characterized by dysplasia that often progress to acute myeloid leukemia (AML). Our goal was to identify genes dysregulated by vector-mediated genotoxicity that may collaborate with the RUNX1 mutant (D171N). D171N expressing cells have a survival and engraftment disadvantage and require additional genetic lesions to survive and persist. By dysregulating genes near the integrated vector provirus, the shuttle vector can promote transformation of D171N cells and tag the nearby genes that collaborate with D171N. In our approach, a gammaretroviral shuttle vector that expresses D171N is used to transduce CD105^+^, Sca-1^+^ mouse bone marrow. Mutagenized cells are expanded in liquid culture and vector integration sites from surviving cells are then identified using a retroviral shuttle vector approach. We repeatedly recovered integrated vector proviruses near genes (*Itpkb*, *Ccdc12*, and *Nbeal2*). To assess the prognostic significance of the genes identified we examined differential expression, overall survival, and relapse free survival of AML patients with alteration in the genes identified using The Cancer Genome Atlas (TCGA) AML data set. We found that ITPKB functions as an independent factor for poor prognoses and RUNX1 mutations in conjunction with ITPKB, CCDC12, and NBEAL2 have prognostic potential in AML.

## INTRODUCTION

Acute myeloid leukemia (AML) is a clonal disorder arising from a heterogeneous population of pre-leukemic and leukemic cells. Investigations of the molecular mechanisms that mediate AML are confounded by tumor heterogeneity, which makes it difficult to identify the causative mutation amongst the multiple background or passenger mutations accumulated in cancerous cells. Several driver genes have now been identified in myeloid leukemogenesis, however many mutations and potential collaborating mutations have yet to be identified. The RUNX1 (AML1) mutant (D171N) occurs in 40% of myelodysplastic syndromes (MDS), with 30%–40% undergoing transformation to AML [1]. The D171N mutation has a survival and engraftment disadvantage and requires additional genetic lesions for mutated cells to survive and persist [1, 2]. Identifying the genes that collaborate with the D171N mutation is important as these genes may have prognostic potential to improve outcomes for MDS/AML patients. With molecular testing entering mainstream clinical practice, identifying new driver genes and contributing factors may improve the ability to screen and diagnose patients [3].

Insertional mutagenesis screens have been used for more than three decades to identify genes involved in oncogenesis [4]. Cells with vector integrations that dysregulate nearby genes that provide a survival advantage become over-represented as they outcompete other cells. The integrated vector provirus acts as a genomic tag and recovering these vector integrations and mapping their genomic locations can identify the nearby genes [5]. Using a gammaretroviral shuttle vector that expresses D171N we attempted to identify gene partners that may collaborate with D171N and demonstrate prognostic potential. Our shuttle vector approach rescues vector provirus junctions with genomic DNA as bacterial plasmids. This in turn allows for rapid mapping of genes near the integrated vector provirus that may be dysregulated by the vector, and collaborate with D171N. Here we report recovery of retroviral insertion sites (RIS) using our shuttle vector rescue approach in liquid cultures of mutagenized murine bone marrow to identify gene candidates that may collaborate with the D171N mutant. The genes identified using our approach are recurrently altered in The Cancer Genome Atlas (TCGA) [6] AML patient data samples and demonstrate prognostic potential.

## RESULTS

### AML1 D171N shuttle vector

**Figure 1:**
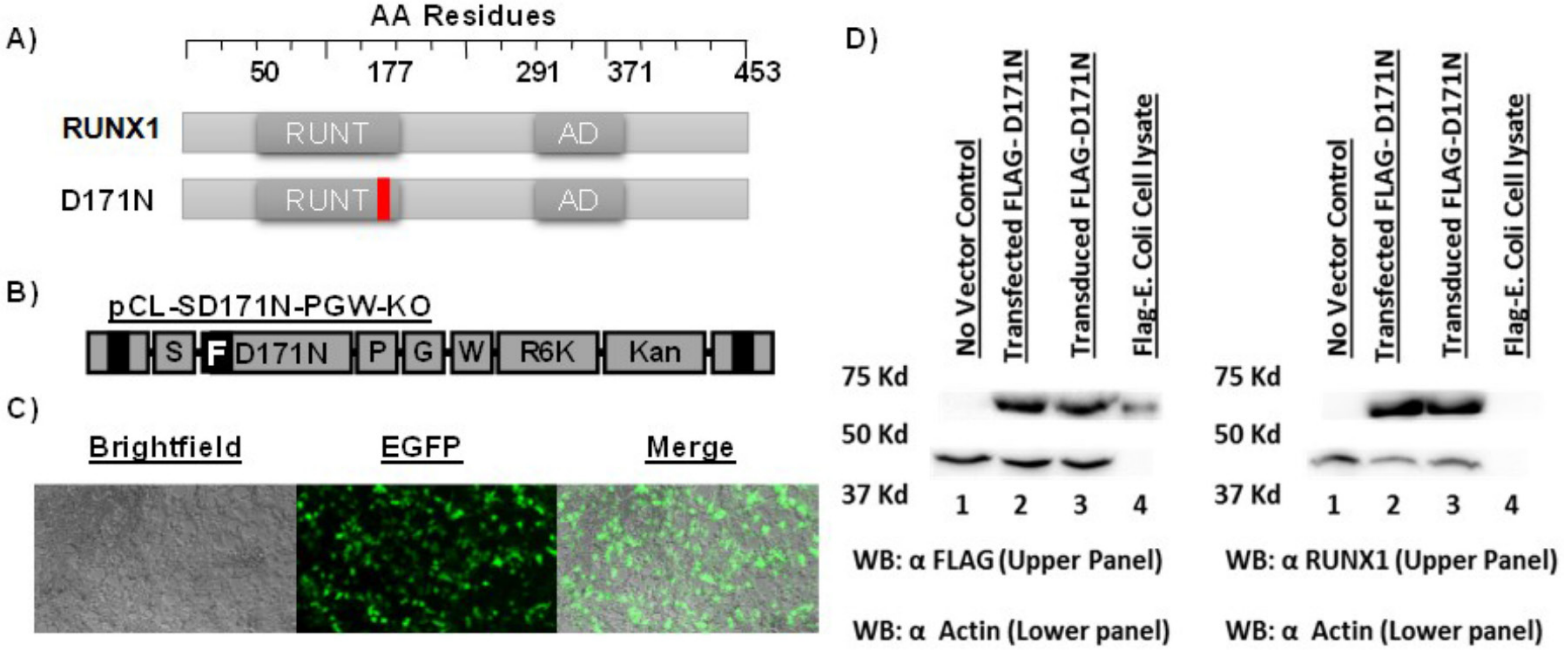
Expression and function of FLAG-AML1 D171N **A.** Schematic of RUNX1 (WT), and D171N mutant. **B.** Schematic of gammaretroviral vector pCL-SD171N-PGW-KO. Flag epitope tagged [F] AML1-D171N is expressed from an internal spleen focus forming viral promoter [S]. Enhanced green fluorescent protein [G] is expressed from a second internal phosphoglycerate kinase-1 promoter [P]. The vector contains a modified woodchuck hepatitis response element [W] for increased stability. The R6Kγ bacterial origin of replication and kanamycin resistance gene [KO] allow for efficient shuttle vector rescue in bacteria. **C.** Transduced HEK293T cells. **D.** Protein expression of mutant D171N is confirmed by Western blot. HEK293T cells do not express AML1b, and serve as a negative control. Positive *E. coli* FLAG protein lysates were used as a positive FLAG epitope control. Actin was used as an equal loading control.

Our goal was to efficiently identify potential collaborating genes and perform a preliminary validation of these gene candidates in patients with AML. Our strategy was to engineer a genotoxic shuttle vector rescue plasmid that expressed the D171N mutant to identify genes that may collaborative via insertional mutagenesis. This vector expresses the D171N protein using the spleen focus forming virus (SFFV) promoter which is known to efficiently dysregulate nearby genes. To validate the expression and function of the mutant D171N protein (Fig. 1A) from the gammaretroviral shuttle vector (Fig. 1B), human embryonic kidney (HEK 293T) cells were transduced (Fig. 1C). Antibodies against FLAG epitope tag and the C terminal domain of D171N were used to confirm expression by western blot analysis. HEK 293T cells do not express endogenous RUNX1 and serve as a negative control. Whole cell lysates of D171N transduced HEK 293T cells demonstrated robust expression of the D171N protein (Fig. 1D) thereby demonstrating the vector could efficiently deliver D171N.

**Figure 2:**
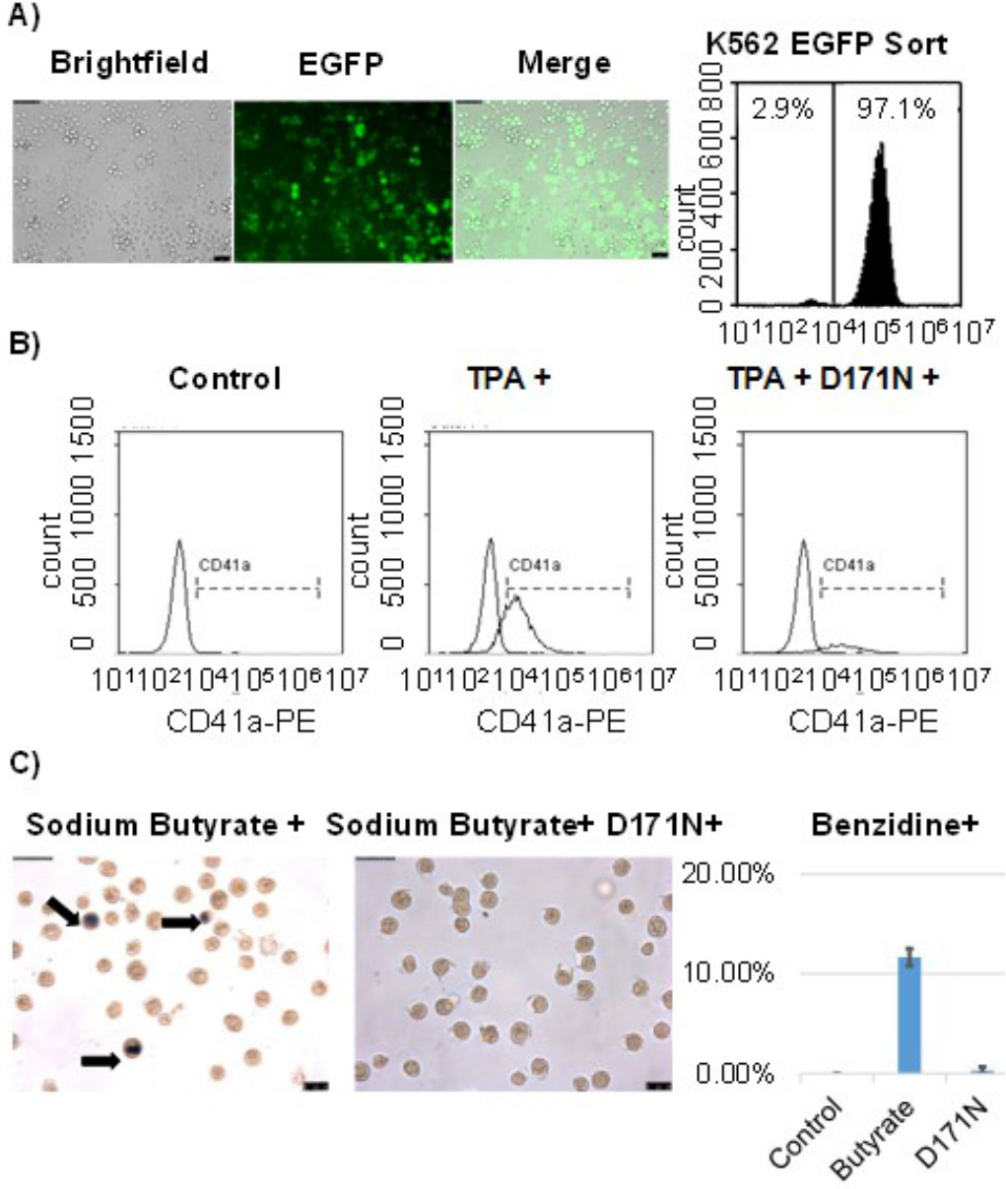
Induced differentiation in K562 cells is blocked by D171N **A.** K562 cells were transduced with pCL-SD171N- PGW3-KO and sorted for purity reaching 97% EGFP positive cells. **B.** K562 cells were induced towards megakaryoblasts with TPA treatment. Differentiation was measured by the expression of megakaryocytic differentiation-specific membrane antigen (CD41a) for control K562, TPA treated K562, and D171N expressing TPA treated K562 cells. **C.** Erythroid differentiation of K562 cells was induced with sodium butyrate. Hemoglobin expression was measured using benzidine staining. Control K562 cells (<1%), Sodium Butyrate treated K562 cells (12%), and D171N Sodium Butyrate treated K562 cells (<1%).

To confirm the function of the dominant negative D171N mutant we performed *12-O-tetradecanoylphorbol-13-acetate* (TPA) and sodium butyrate induced differentiation assays in the erythroleukemia cell line K562. K562 cells were transduced and sorted for EGFP expression prior to the assay (Fig. 2A). Cells treated with TPA readily differentiated into megakaryocytic blasts as determined by flow cytometric analysis using the megakaryocytic marker CD41a (Fig. 2B). K562 cells treated with sodium butyrate differentiated into erythrocytes, as detected by hemoglobin expression measured with benzidine staining (Fig. 2C). Cells transduced with the shuttle vector expressing D171N demonstrated reduced differentiation after stimulation with TPA and sodium butyrate, as evidenced by flow cytometry and benzidine staining (Fig. 2B, 2C). These results demonstrate the dominant negative function of mutant D171N in blocking TPA and sodium butyrate induced differentiation of K562 cells. Furthermore, D171N transduced cells demonstrated reduced cell viability with trypan blue staining during expansion for differentiation assays demonstrating a survival disadvantage (data not shown). This is consistent with others reports that D171N induces cell cycle arrest and increases apoptosis [2, 7].

### Transduction of mouse bone marrow with D171N shuttle vector

**Figure 3:**
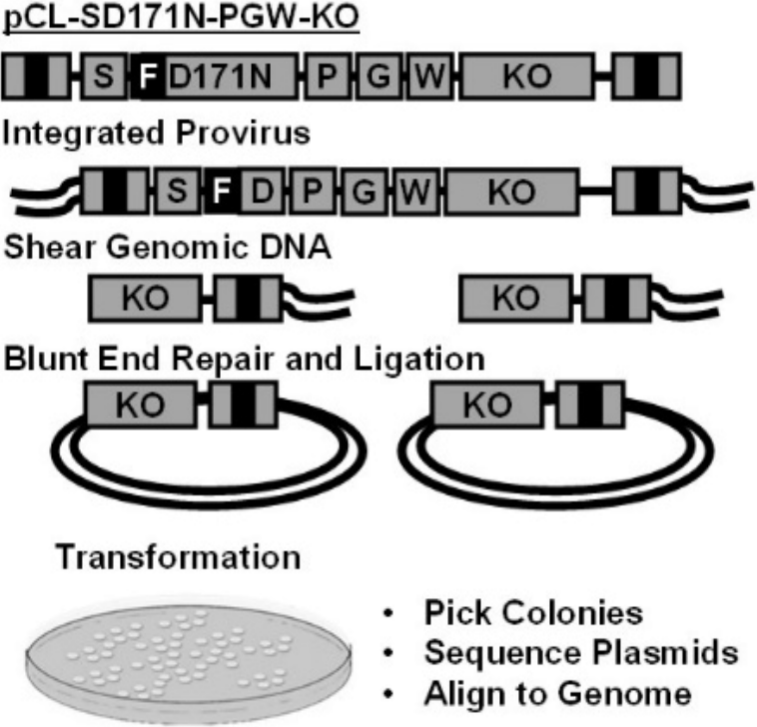
Shuttle vector rescue Replication incompetent gammaretroviral vector pCL-SD171N-PGW3-KO. Genomic DNA containing vector provirus is sheared, forming genomic DNA fragments containing the R6Kγ origin of replication, kanamycin resistance gene (KO), and viral LTR B). Shuttle vector rescue of genomic plasmids grown in *E. coli*, picked, and sequenced for vector-chromosome junctions using a 3′ LTR specific primer. Sequences are trimmed removing the LTR and aligned to the human genome to identify integration sites.

Enriched murine CD105^+^, Sca-1^+^ cells (1 × 10^6^) were recovered with cytokine support before exposure to the vector expressing FLAG tagged D171N (Fig. 1A, 1B). To determine the viability of enriched CD105^+^, Sca-1^+^ bone marrow cells, replicates of 2×10^4^ transduced cells were plated in semi-solid media for colony forming unit (CFU) assays which showed that vector exposed cells readily formed CFUs. The remaining cells were maintained in liquid culture for expansion and retrieval of retroviral integration site using a shuttle vector approach (Fig. 3). Marking of mutagenized cells reached 1~5% in liquid cultures (5×10^4^ cells) based on flow cytometric analysis, and 5% in CFU assays, demonstrating transduction of enriched CD105^+^, Sca-1^+^ cells. Transduction efficiencies were lower than expected, and were limited by the titer of our large gammaretroviral shuttle vector.

### RIS analysis from liquid cultures of mutagenized hematopoietic progenitors

**Figure 4:**
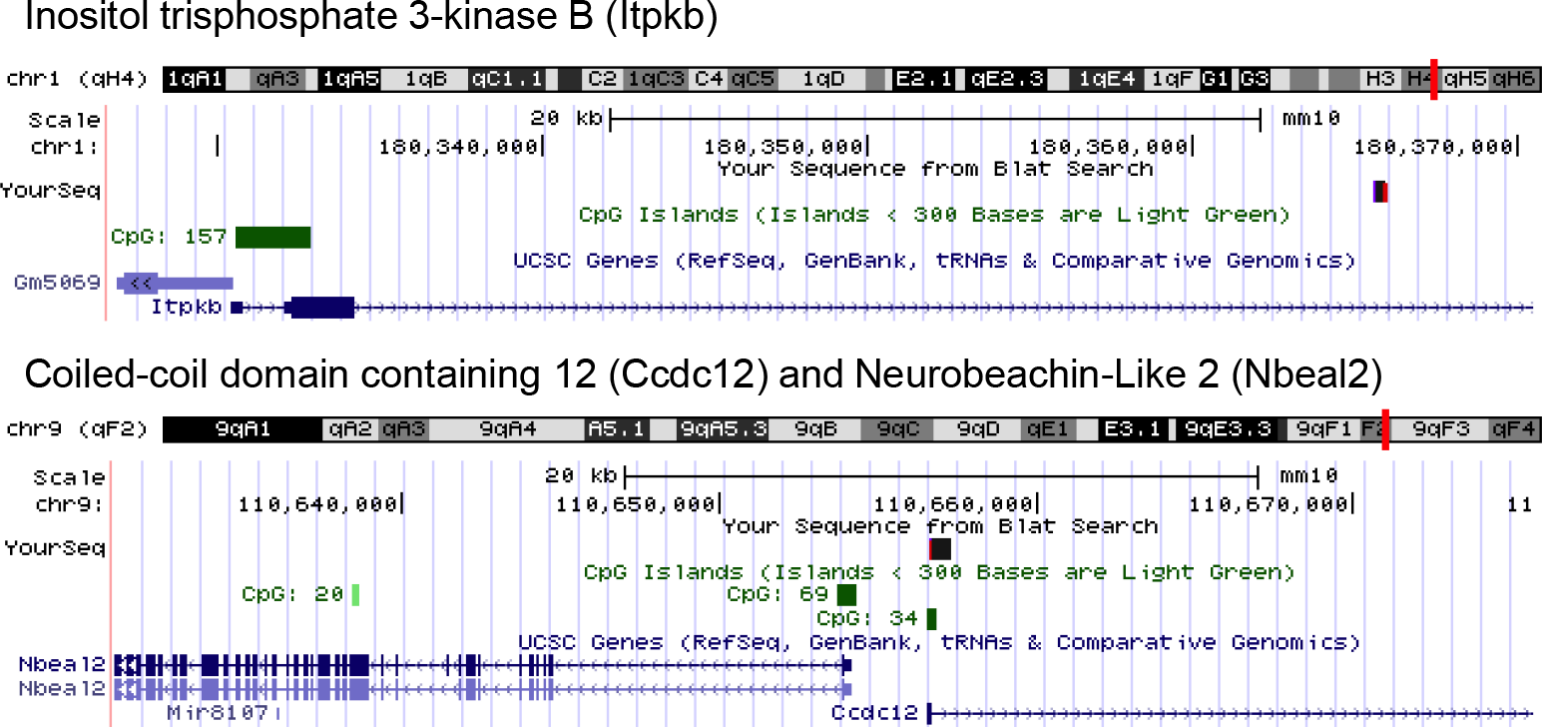
Vector-chromosome junctions Vector-chromosome junctions were mapped to the mouse genome using the UCSC BLAT genome browser. The first identified integration occurred within the inositol triphosphate 3-Kinase B (*Itpkb*) gene. The second integration occurred within a CpG island between gene regions for Coiled-coil domain containing 12 (*Ccdc12*) and Neurobeachin-like2 (*Nbeal2*).

Our approach was to expand mutagenized cells in liquid culture to identify potential clonal outgrowth. Mutagenized liquid cultures of murine CD105^+^, Sca-1^+^ cells were expanded for 14 days to allow for clonal selection and outgrowth or skewing to occur. The liquid cultures were then lysed and genomic DNA harvested for shuttle vector rescue and integration analysis was performed using VISA software [8] (Sup. Table 1). Analysis of integrations recovered from CD105^+^, Sca-1^+^ mutagenized cells identified three highly represented gene candidates (Fig. 4). One of the RIS identified occurred within the gene inositol triphosphate 3-kinase B (*Itpkb*). Another RIS occurred within a CpG island between two neighboring genes Coiled-coil domain containing 12 (*Ccdc12*), and Neurobeachin-like2 (*Nbeal2*). (Fig. 4).

### Gene candidates are prognostic factors of AML

**Figure 5:**
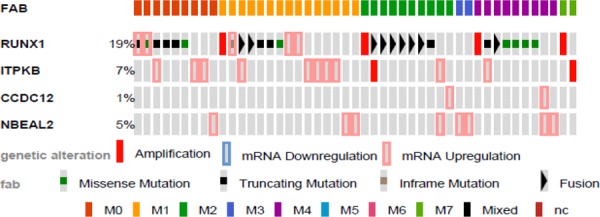
Genetic alterations of identified genes in human AML by FAB Identified genes were analyzed using cBioPortal on 166 fully annotated patients from the 2013 TCGA human AML data set. The percentage of total altered cases in the data set is listed, and only cases with genetic alterations are shown. Cases are sorted by the French, American, and British (FAB) subtype.

We were interested to determine if *ITPKB*, *CCDC12*, and *NBEAL2* may play a prognostic role in human AML. We first determined if these genes were commonly altered in AML patients. To perform this analysis we used cBioPortal for Cancer Genomics, which provides visualization and analysis tools for large-scale cancer genomics data sets containing clinical information, expression data, and molecular data. The 2013 TCGA AML data set contained 166 fully annotated AML cases, which we queried for the gene candidates found in our screen. *RUNX1*, *ITPKB*, and *NBEAL2* were frequently mutated in patient samples (Fig. 5).

Mutations were analyzed for mutual exclusivity, with a trend of co-occurrence between *RUNX1* and *ITPKB* alterations (Sup. Table 2). Using SurvExpress, an online biomarker validation tool and database for cancer gene expression on the 2013 TCGA AML leukemia data set, we observed significant differential expression of all genes identified using our approach in high and low risk AML (Sup. Fig. 1).

**Figure 6:**
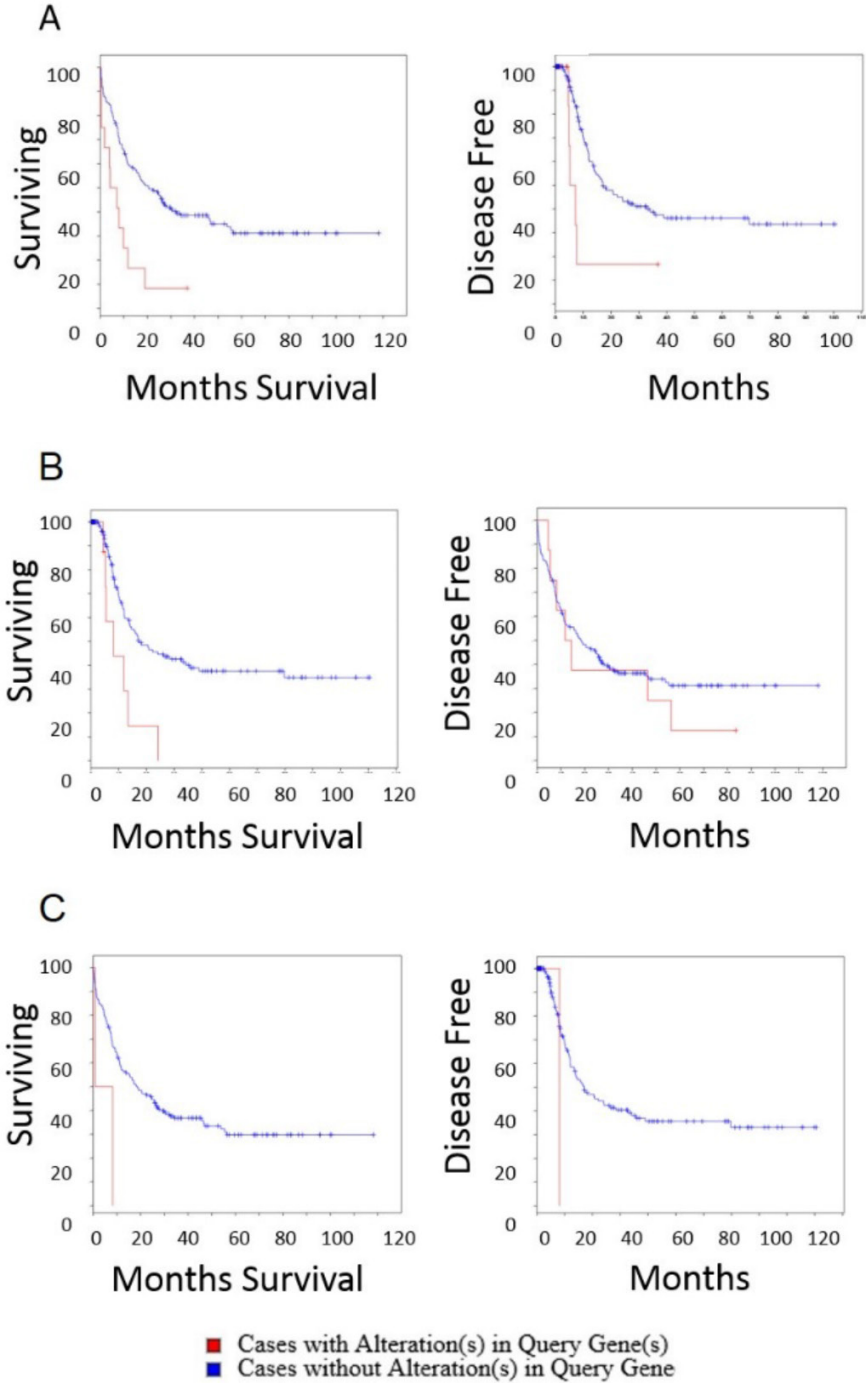
Overall survival and disease free survival analysis of ITPKB, NBEAL2 and CCDC12 in AML Left panels are overall survival and right panels are disease free survival after initial treatment **A.** ITPKB. Overall survival time for patients that do not harbor alterations in ITPKB is significantly longer than patients with alterations in ITPKB (log rank test *P*-Value: 5.5 × 10^−4^) (Cases with alteration in query gene: total cases: 12, deceased: 5, median months survival: 5.2, cases without alteration in query gene: total cases: 154, deceased: 98, median months survival: 20.5). Patients with ITPKB alterations had significantly lower disease free survival (log rank test *P*-Value: 0.01) (Cases with alteration in query gene: total cases: 12, deceased 5 median months survival 5.2, Cases without alteration in query gene: total cases: 152, deceased: 72, median months survival: 17). **B.** NBEAL2. The overall survival time for patients that do not harbor alteration in NBEAL2 is significantly longer than patients with alterations in NBEAL2 (log rank test *P*-Value: 0.004) (Cases with alteration in query gene: total cases: 8, deceased: 11, median months survival 11.8, cases without alteration in query gene: total cases: 154, deceased: 98, median months survival: 18.5). Effect of NBEAL2 alteration on disease free survival after initial treatment. Disease free survival was not significantly different for patients with or without NBEAL2 alterations (log rank test *P*-Value: 0.53) (Cases with alteration in query gene: total cases: 2, deceased: 1, median months survival: 8.2, cases without alteration in query gene: total cases: 162, deceased: 76, median months survival: 17). **C.** CCDC12. The overall survival time for patients that do not harbor alteration in CCDC12 is significantly longer than patients with alterations in CCDC12 (log rank test *P*-Value: 0.03) although there were few patients harboring this mutation in the 2013 TCGA cohort (Cases with alteration in query gene: total cases: 2, deceased: 2 median months survival: 0.8, cases without alteration in query gene: total cases: 164, deceased: 107, median months survival: 18.5). The disease free survival was not significantly different for patients with or without CCDC12 alterations (log rank test *P*-Value: 0.18) (Cases with alteration in query gene: total cases: 2, deceased: 1, median months survival: 8.2, cases without alteration in query gene: total cases: 162, deceased: 76, median months survival: 17).

With the gene alterations recurring in many AML patients, and differential expression between high and low risk AML, we next investigated whether the genes identified could stratify patients for overall survival and disease free survival (Fig. 6). All three candidate genes when altered in patients demonstrated significantly lower overall survival rates. Interestingly *ITPKB* was also prognostic for disease free survival, though *CCDC12* and *NBEAL2* did not reach statistical significance. This may have been a result of the small number of patients in the TCGA data set harboring *CCDC12* and *NBEAL*2 alterations.

**Figure 7:**
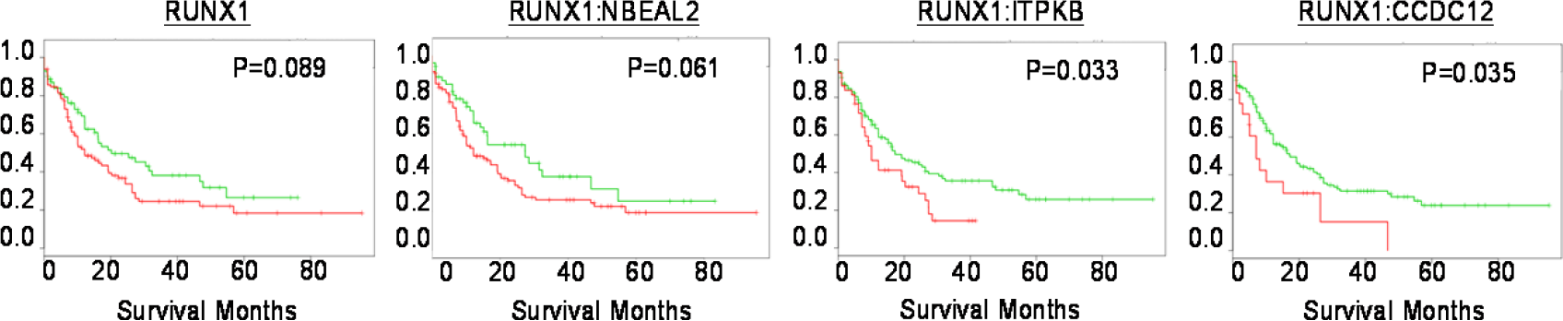
The expression of identified genes predict patient survival Kaplan-Meier analysis conducted with SurvExpress. Analysis of RUNX1 status alone, or in conjunction with genes identified in our screen (*NBEAL2*, ITPKB*, CCDC12*). *p = < 0.05

Using SurvExpress, we performed differential expression analysis using the 2013 AML TCGA cancer database to predict patient survival outcomes. Comparisons were performed with *RUNX1*, and combinations of *RUNX1* with potential collaborator genes identified in our study (Fig. 7). Two genes identified in our study (*ITPKB*, *CCDC12*) demonstrated significant stratification of low and high risk AML patient survival (*P* = 0.03 *CI* = 55), with higher significance than *RUNX1* status alone (*P* = 0.08 *CI*=52).

**Figure 8:**
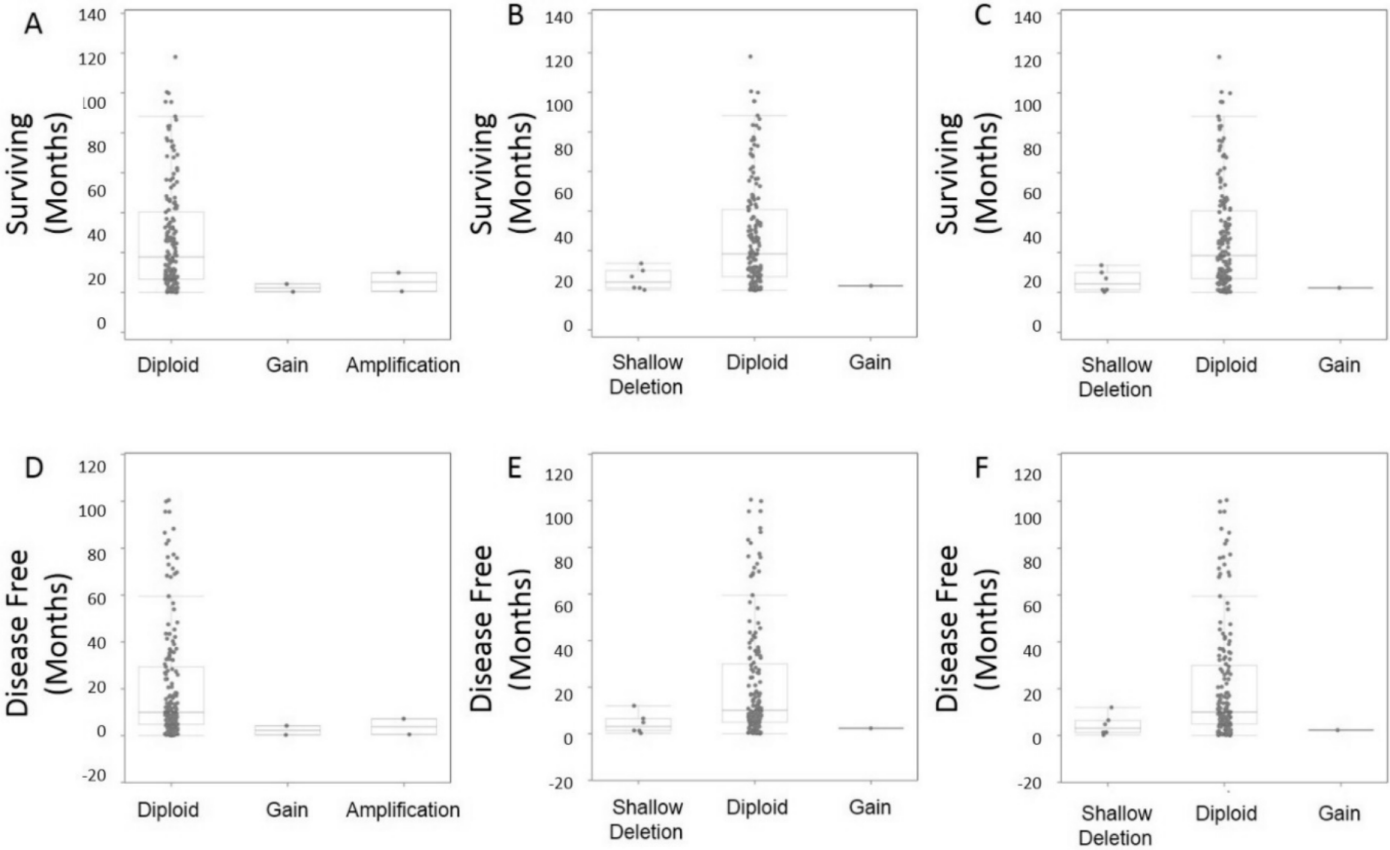
CNA survival analysis Copy number alterations were observed for overall survival and also disease free survival post initial diagnosis/treatment **A.** CNA ITPKB demonstrated reduced survival in patients with gains or amplifications. **B.** CNA CCDC12 demonstrated reduced survival in patients with shallow deletions. **C.** CNA NBEAL2 demonstrated reduced survival in patients with shallow deletions. **D–F.** Disease free survival was also explored by CNA demonstrating all gene alterations (ITPKB, CCDC12, NBEAL2) lead to higher incidence of disease recurrence.

Differential expression of the candidate genes lead us to examine copy number abnormalities (CNA) since there were no recorded point mutations for *ITPKB*, *CCDC12*, and *NBEAL2* in the 2013 TCGA patient molecular sequencing data set. Analysis of overall survival and disease free survival by CNA, showed that gain or large amplifications of *ITPK*B corresponded to significantly (*P* Value 5.5 × 10^−4^) higher rates of death with median survival of *ITPKB* alteration at 8.2 months compared to no alterations at 20.5 months (Fig. 8). For *CCDC12* and *NBEAL2* shallow deletions or loss of a single allele were associated with significant (*P* Values: 0.004, and 0.03) rates of death compared to patients without gene alterations (Fig. 8). Median survival for patients with alterations in *NBEAL2* and *CCDC12* (11.8 0.8 months) compared to patients without alterations (18.5 months) respectively.

## DISCUSSION

MDS/AML is highly heterogeneous, and identifying the oncogenic drivers and collaborators has prognostic and therapeutic significance [9]. Here we have established a novel gammaretroviral shuttle vector approach to rapidly identify potential gene collaborators in the pathology of D171N AML. RUNX1 mutations are the most frequently occurring mutations in MDS/AML [7]. The D171N point mutation occurs within the RUNT DNA binding domain, which hinders binding in the minor groove of RUNX1 target sequences. Functionally, D171N acts in a dominant negative fashion, blocking myeloid differentiation, and greatly reduces cell survival in the absence of collaborating mutations. D171N also is reported to abrogate engraftment potential and induce cell cycle arrest and apoptosis [2]. This is consistent with our findings in a murine bone marrow transplantation model, and those reported in leukemic cell lines [7]. The engraftment and survival disadvantage means that cells with the D171N mutation need mutations in genes that collaborate to promote cell survival and expansion. Thus we used *ex vivo* culture to expand mutagenized enriched bone marrow cells, and collected genomic DNA to identify gene candidates that may collaborate with D171N.

Previous studies have shown that the D171N effects on engraftment and survival can be rescued through strong, independent collaborating mutations in genes such as *Evi-1* and *Bmi1* [2, 10]. In the two hit model of leukemogenesis, class I or proliferation mutations must be acquired in conjunction with Class II differentiation block mutations such as D171N for cell survival and leukemic transformation [11]. Cells harboring integrations near genes that provide a survival advantage will expand in liquid culture, allowing for their efficient rescue with our shuttle vector approach.

Here we identified potential D171N novel collaborators (*ITPKB*, *CCDC12*, and *NBEAL2*). These three genes appear to be involved in AML pathogenesis. All three candidates identified using our approach were recurrently altered in AML patients, and demonstrated differential expression between high and low risk AML. This led us to explore ITPKB, CCDC12, and NBEAL2 as prognostic factors for AML.

ITPKB has appeared in previous screens for phosphoinositide modulators and leukemia, [12–14] and has been described in B cell development and function [15, 16], and also as a master regulator of hematopoietic stem cell homeostasis [17]. These reports suggest that ITPKB acts differently in a cell type specific manner, and may be a negative regulator of leukemia [13]. Interestingly in the 2013 TCGA AML patient expression data, thecBioPortal gene alteration analysis showed the mRNA for ITPKB is upregulated in AML patients (Sup. Fig. 1) and may be AML intrinsic.

Using knockdown of ITPKB, Jude et al. [13] demonstrated increased cellular expansion of specific AML cell lines. The cellular expansion resulting from loss of ITPKB was also demonstrated in hematopoietic stem cells deficient for ITPKB [17]. Integration of vector provirus within the *ITPKB* gene may have induced vector mediated enhancer activation of the *ITPKB* gene or could have inactivated one allele resulting in haploinsufficiency, accounting for expansion of this clone, and thus recovery of this integration site from expanded liquid cultures. The mechanisms and interplay between ITPKB and D171N will need further investigation. Recent work has shown that ITPKB is recurrently altered in human cancers and represents a significant regulator of AML cell proliferation and survival, though alone ITPKB dysfunction does not appear to promote leukemic transformation [13, 17]. ITPKB may also serve as a therapeutic target via regulation of RASA3, with rapamycin treatment in AML patients [15, 16]. Here we report that the *ITPKB* gene identified in our retroviral mutagenesis screen is an independent prognostic factor in AML. This finding is important clinically as ITPKB expression has also been identified in resistance to therapy and may serve to stratify patient treatment modalities [18].

Our data are the first to our knowledge to suggest ITPKB may collaborate with D171N. ITPKB knockdown acts as a strong class I gene that could potentially collaborate with the D171N class II mutation. Network analysis demonstrates that RUNX1 and ITPKB share interactions (Sup. Fig. 2) and suggest that additional studies to confirm the role of ITPKB in MDS/AML are warranted.

The genes Coiled-coil domain containing 12 (*CCDC12*) and Neurobeachin-Like 2 (*NBEAL2*) may also be potential collaborators, though the evidence is lacking in our report due to the few number of altered cases in the 2013 TCGA AML patient data set (2, and 8 of 166 patients). Analysis of gene expression in patients for CCDC12 demonstrated differential expression between high and low risk AML, and predicted patient overall survival.

Fan et al. identified CCDC12 as a new participant that promotes early erythroid differentiation [19]. Overexpression of CCDC12 in K562 cell lines demonstrated an increased growth advantage. While the role of CCDC12 in MDS/AML remains unknown, our study is the first to identify *CCDC12* as a potential collaborating gene with D171N and suggest the gene would be an excellent candidate for follow on studies. Furthermore, the *NBEAL2* gene which is located near *CCDC12* is already recognized in familial platelet disorders with a predisposition to MDS/AML [20, 21].

In summary, our D171N retroviral shuttle vector mutagenesis screen identified three genes, *ITPKB*, *CCDC12*, *and NBEAL2 in* human AML. The candidate genes we identified are prognostic factors in AML, and here we report for the first time to our knowledge that *ITPKB* is an independent prognostic factor for overall survival and disease free survival in AML patients. ITPKB may also serve to stratify patients to specific treatment modalities as alterations in ITPKB have now been linked to specific drug resistance [18]. Our shuttle vector approach has broad applications for identifying potential gene candidates in other leukemia and cancer settings.

## MATERIALS AND METHODS

### Mouse bone marrow harvest and culture

Hematopoietic mononuclear cells were isolated from the femurs of B6.SJL-Ptprc^b^ (Ly5.1) congenic mice (9–12 weeks of age) 4 days after intraperitoneal administration of 150 mg/kg 5-fluorouracil (5-FU). Fluorescence activated cell sorting of CD105^+^, Sca-1^+^ labeled cells was used to enrich samples [22–24]. 1 × 10^6^ Ly5.1 enriched bone marrow cells were then cultured as previously described [10]. The pre-stimulated cells were plated in 6-well dishes coated with CH-296 murine fibronectin fragment (TAK T100A, TAKARA Bio, Shiga, Japan) at 2 μg/cm^2^ and exposed to vector at a multiplicity of infection of 5 for 24–72 hours.

### Shuttle vector plasmid construction

The human RUNX1 (AML1) mutant D171N identified previously from patient case no. 5 [25] was synthesized by Blue Heron Bio, LLC (Bothell, WA) with the D171N point mutation to the published AML1b sequence (NCBI: NM_001001890.2) and codon optimized for murine expression. The D171N protein was produced with a modified 5′ FLAG epitope tag with a 6-G-polylinker on the N-terminus (Sup. Fig. 3). A spleen focus forming viral promoter (SFFV) drives D171N gene expression. A human phosphoglycerate kinase-1 (PGK) promoter drives enhanced green fluorescent protein (EGFP). The gammaretroviral shuttle vector pCL-SD171N-PGW3-KO was generated from pCAG-GFP, a gift from Fred Gage (Addgene plasmid # 16664) [26] by cloning synthesized D171N expression cassette and also the shuttle rescue cassette from LV-SFFVEGFP [27] using standard molecular biology techniques. Shuttle vector rescue cassette encodes a bacterial origin of replication R6Kγ, and kanamycin resistance gene for recovery of vector integration sites in bacteria after sequencing for LTR: chromosome junctions.

### Vector production

Gammaretroviral vector production was carried out in HEK293T cells maintained in Dulbecco's modified eagle medium (DMEM) supplemented with 10% fetal bovine serum (FBS) and transfected with the gammaretroviral vector plasmid pCL-SD171N- PGW3-KO, and helper plasmids pLGPS, and pMD2.G. Medium was changed 24 hours after transfection, and supernatant harvested at 48 hours. Pooled virus-containing medium was filtered with 0.45 μM filter and centrifuged for 18 hours at 8,000 g. Viral vector pellets were resuspended in culture medium at 1/100 of the initial volume. Vector titers were determined using HT1080 fibrosarcoma cells and analyzed by flow cytometric analysis for EGFP expression.

### Western blot analysis of D171N function

HEK293T cell lysates were resolved by SDS-PAGE on 8% acrylamide gels, transferred to polyvinylidene difluoride membrane, probed with specific antibodies and detected by enhanced chemiluminescence on a BioRad Chemi Doc XSR+ imager. Rabbit α-mouse AML1 (clone EPR3099, LifeSpan Biosciences, Seattle, WA), Rabbit α-FLAG (clone OctA-Probe D:8 sc-807 HRP, Santa Cruz Biotechnology, Santa Cruz, CA), Rabbit β-actin (23277: 600-401-886, Rockland Immunochemicals Inc., Gilbertsville, PA), and Goat α-Rabbit-HRP (Ab97051, Abcam plc., Cambridge, MA) were used.

### K562 differentiation assay

K562 cells were sorted for EGFP expression on an S3 cell sorter (BioRad, Hercules, California). Erythroid differentiation of K562 cells was induced 3 days after vector exposure by plating at 2 × 10^5^ cells/mL and culturing in media supplemented with 0.6 nM sodium butyrate for 3 days. Megakaryoblastoid differentiation of K562 cells was induced 3 days after vector exposure by plating 2 × 10^5^ cells/mL and culturing in media supplemented with 50 nM TPA for 3 days. After 6 days of culture, cells were assayed for megakaryocytic differentiation by measuring the expression of differentiation-specific membrane antigen (CD41a) [28]. Cells were washed and suspended in ice-cold PBS containing 10% fetal calf serum. Anti-CD41a-PE Ab was added for 30 min at 4°C and the cells were then washed twice with 2 ml of PBS containing 10% fetal calf serum. CD41a (ab19690, Abcam plc. Cambridge, MA) expression was evaluated via flow cytometric analysis (AccuriC6, BD). Benzidine stock solution contained 0.2% w/v benzidine hydrochloride in 0.5 M acetic acid. 1 × 10^5^ cells were washed twice with ice-cold phosphate-buffered saline. The cell pellets were resuspended in ice-cold phosphate-buffered saline (27 μl). The benzidine solution (3 μl) containing hydrogen peroxide (final concentration, 0.0012%) was added and incubated for 10 minutes at room temperature. Benzidine-positive cells were quantitated by light microscopy. At least 100 cells were counted in triplicate for each condition.

### Hematopoietic progenitor assay

One day post vector exposure, 2×10^4^ cells were plated in semisolid MethoCult™ methylcellulose media (M3234, Stemcell Technologies, Vancouver, BC, Canada). MethoCult™ was prepared according to manufacturer's directions, and the following cytokines were added: 50 ng/mL of mouse stem cell factor (SCF), mouse Fms-like tyrosine kinase 3 ligand (FL), human IL-6, and human thrombopoietin (TPO); (R&D Systems, Minneapolis, MN). Methocult™ plates were incubated at 37°C. Fourteen days later, CFUs were counted and scored for EGFP expression using fluorescence microscopy in triplicates. Plating efficiency was calculated as CFUs/cells plated x 100.

### 14 Day liquid culture of enriched mouse CD105^+^ Sca-1^+^ bone marrow

CD105^+^ Sca-1^+^ enriched bone marrow culture was initiated with 1 × 10^6^ cells per well of a 6 well plate. When cells reached confluence they were cultured in a 10 cm plate until harvest at day 14. Cells were maintained in StemSpan^TM^ media (StemSpan serum-free expansion medium, Stemcell Technologies, Vancouver, BC, Canada) supplemented with recombinant human fibroblast growth factor 1 (10 ng ml^−1^), mouse thrombopoietin (50 ng ml^−1^), mouse insulin growth factor II (20 ng ml^−1^) and mouse stem cell factor (20 ng ml^−1^); (R&D Systems, Minneapolis, MN). Cells were incubated at 37°C. Fourteen days later, cells were collected for flow cytometric analysis of EGFP and genomic DNA isolation for shuttle vector rescue.

### Shuttle vector rescue analysis

Genomic DNA was isolated from mouse bone marrow using Qiagen Puregene cell and tissue kit (158388, Qiagen, Valencia, CA) and 3 μg DNA randomly sheared using a fluidic hydroshear device (Digilab Inc., Marlborough, MA). Sheared genomic DNA fragments were end repaired (blunt ended) and ligated to form rescue plasmids using DNATerminator^®^ End Repair Kit (Lucigen Corp., Middleton, WI). Genomic plasmids were then transformed into *E. cloni* G, (Lucigen Corp., Middleton, WI) and grown on kanamycin plates. Kanamycin resistant colonies were sequenced by HtSeq (University of Washington HtSeq, Seattle, WA) using gammaretroviral LTR1 primer (5′-CTTGTGGTCTCGCTGTTCCTTGG-3′). Sequences were analyzed using the vector integration site analysis (VISA) [8] and UCSC BLAT software [29–31] to identify the location of the viral integration site.

### Meta-analysis of genes in patients

cBioportal of cancer genomics was used to examine the genetic alteration of the candidate genes in AML patient samples using the 2013 AML TCGA data set with 166 fully sequenced tumors [32, 33]. Genes identified in our retroviral mutagenesis screens were also evaluated for biomarker potential and risk assessment in patient samples from publically available cancer databases using SurvExpress [34].

## SUPPLEMENTARY FIGURES AND TABLES


