# Supplementary material for: A novel retroviral mutagenesis screen identifies prognostic genes in RUNX1 mediated myeloid leukemogenesis
Source: Oncotarget. 2015 Sep 12;6(31):30664–74. doi: 10.18632/oncotarget.5133 (PMC4741560; doi:10.18632/oncotarget.5133)
Supplement: Supplementary file 1 [file oncotarget-06-30664-s001.pdf]

SUPPLEMENTARY FIGURES AND TABLES

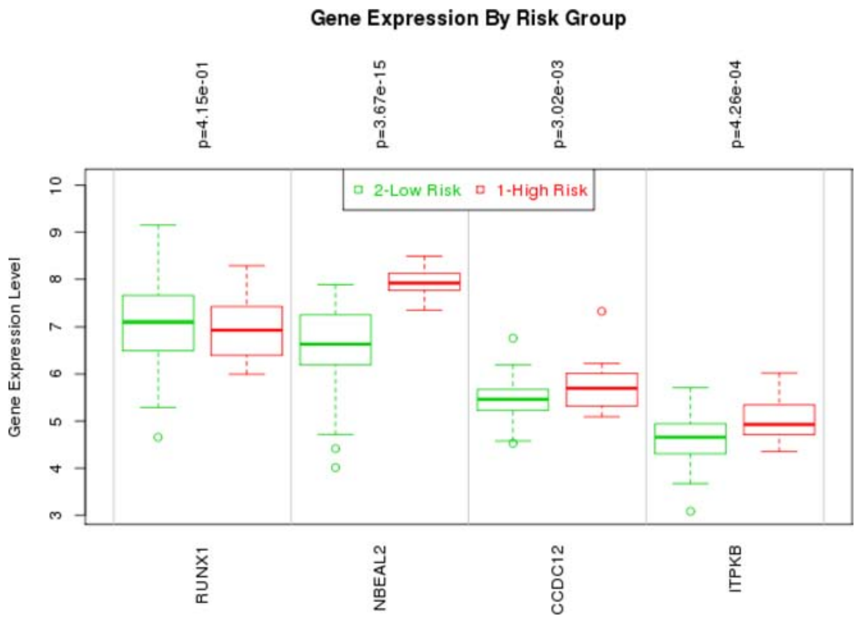

Supplementary Figure S1: Differential Gene Expression in High and Low Risk Acute Myeloid Leukemia. Differential expression of RUNX1,CCDC12, NBEAL2, and ITPKB in high risk and low risk acute myeloid leukemia. Plot was created using SurvExpress with the TCGA Acute Myeloid Leukemia dataset.

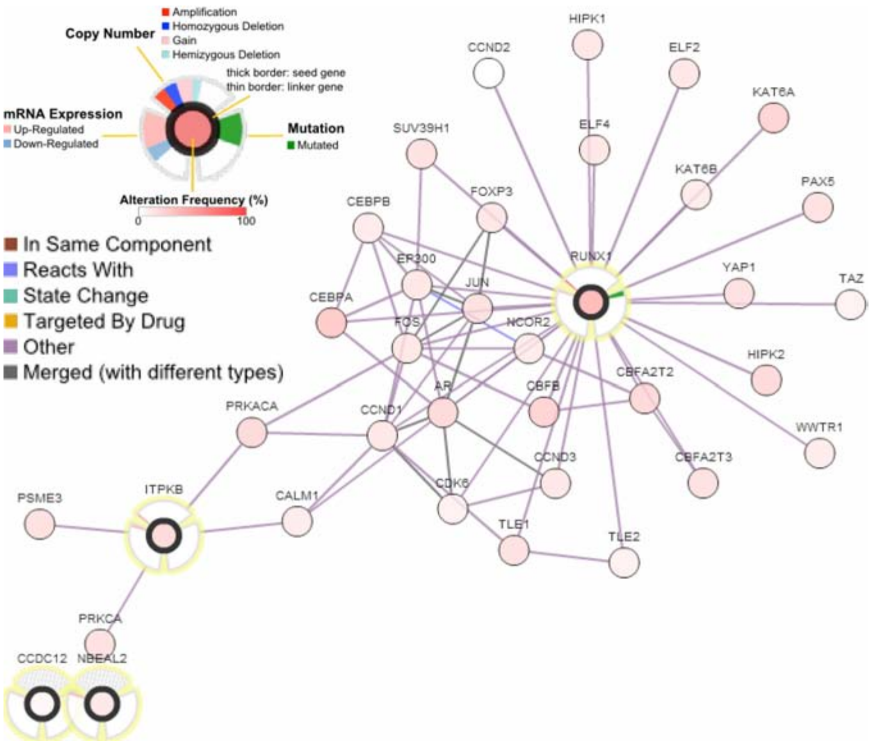

Supplementary Figure S2: Network Analysis of Identified Genes. Network analysis using cBioPortal shows interactions between RUNX1, and ITPKB. CCDC12 and NBEAL2 were not implicated in any known interaction pathways.

5’DYKDDDDK_GGGGGG_MRIPVDASTSRRFTPPSTALSPGKMSEALPLGAPDAGAALAGKLR
SGDRSMVEVLADHPGELVRTDSPNFLCSVLPTHWRCNKTLPIAFKVVALGDVPDGTLVTVMAG
NDENYSAELRNATAAMKNQVARFNDLRFVGRSGRGKSFTLTITVFTNPPQVATYHRAIKITVNG
PREPRRHRQKLDDQTKPGSLSFSERLSELEQLRRTAMRVSPHHPAPTPNPRASLNHSTAFNP
QPQSQMQDTRQIQPSPPWSYDQSYQYLGSIASPSVHPATPISPGRASGMTTLSAELSSRLSTA
PDLTAFSDPRQFPALPSISDPRMHYPGAFTYSPTPVTSGIGIGMSAMGSATRYHTYLPPPYPGS
SQAQGGPFQASSPSYHLYYGASAGSYQFSMVGGERSPPRILPPCTNASTGSALLNPSLPNQS
DVVEAEGSHSNSPTNMAPSARLEEAVWRPY 3’

Supplementary Figure S3: AML1 D171N Flag Epitope Tag Protein Sequence. FLAG epitope tag (yellow), with small linker (Green), and AML1b (Blue) and D171N point mutation (Red).)

Supplementary Table S1: RIS recovered from mutagenized cultures

Site	Days after infection	Mouse cr	Blastbit score	Candidate gene
1	14	1	725	Itpkb
2	14	9	1456	Ccdc12

Three independent genomic shears were performed for shuttle vector rescue analysis. Colonies were sent for sequencing with gammaretroviral specific LTR primer. VISA analysis of the 21 colonies identified two integration sites occurring in mouse chromosome 1 and 9. The Blastbit score indicates quality of alignment of the integration query with the genome. Gene analysis using UCSC Blat genome browser identified Itpkb, Ccdc12, and Nbeal2 as candidate genes.

Supplementary Table S2: Mutual exclusivity and Co-occurrence of identified genetic alterations

GeneA	GeneB	*p*-Value	Log odds ratio	Association
ITPKB	RUNX1	0.048763	0.291806	Tendency Towards co-occurrence
ITPKB	CCDC12	0.87206	<–3	Tendency toward mutual exclusivity
ITPKB	NBEAL2	0.402676	0.745333	Tendency Towards co-occurrence
RUNX1	CCDC12	0.770641	<–3	Tendency toward mutual exclusivity
RUNX1	NBEAL2	0.346606	<–3	Tendency toward mutual exclusivity
CCDC12	NBEAL2	0.079768	3.29053	Tendency Towards co-occurrence

Analysis of genes identified in our screen using cBioPortal demonstrated tendencies towards co-occurrence or mutual exclusivity.
